# ASSESSING FRAILTY USING THE FIT-FRAILTY APP IN A NONGERIATRIC PRACTICE: A FEASIBILITY STUDY

**DOI:** 10.1093/geroni/igad104.2807

**Published:** 2023-12-21

**Authors:** Alexa Kouroukis, Suleman Tariq, Jonathan Adachi, George Ioannidis, Courtney Kennedy, Carolyn Leckie, Alexandra Papaioannou, Isabel Rodrigues

**Affiliations:** McMaster University, Hamilton, Ontario, Canada; McMaster University, Hamilton, Ontario, Canada; McMaster University, Hamilton, Ontario, Canada; GERAS Centre for Aging Research, Hamilton, Ontario, Canada; McMaster University, Hamilton, Ontario, Canada; St. Joseph’s Healthcare Hamilton, Hamilton, Ontario, Canada; McMaster University, Hamilton, Ontario, Canada; McMaster University, Hamilton, Ontario, Canada

## Abstract

Frailty is a common medical condition with a prevalence of 24% in adults ≥50 years when using the Frailty Index. Thus, assessing frailty is a priority. The Fit-Frailty Application (App) is a user-friendly and validated measure that incorporates disease-related, physical, cognitive, psychosocial, and functional aspects of frailty. The purpose of this study was to determine the feasibility of using the App in a non-geriatric clinic. We conducted a cross-sectional study in a rheumatology clinic in Hamilton, Ontario. We included participants ≥50 years with osteoporosis who understood English or attended with a caregiver. Our primary outcome was feasibility defined by recruitment rate (criteria for success 90%), length of time to complete the App by a non-healthcare professional (≤15 minutes), and safety/challenges of using the App. Our secondary outcome was to conduct an exploratory analysis between osteoporosis management (osteoporosis medication, vitamin D and calcium) and total Fit-Frailty score. Thirty participants were approached during a routine clinic visit and 25 agreed to participate (mean age 72.2±11.2; 88% female; 44% had higher education). The mean Fit-Frailty score was 0.24±0.14; scores ≥0.25 indicate frailty. Five chose not to participate citing other time commitments. The mean time to complete the App was 15.48±6.6 minutes with no adverse events. Challenges included the need for a private room and space to perform the gait assessment. We found no association between osteoporosis management and Fit-Frailty score (p>0.05). Despite not meeting our feasibility criterion for recruitment, the App was a feasible tool to measure frailty in a non-geriatric clinic.

